# The Effect of Rapport on Data Quality in Face-to-Face Interviews: Beneficial or Detrimental?

**DOI:** 10.3390/ijerph182010858

**Published:** 2021-10-15

**Authors:** Melany Horsfall, Merijn Eikelenboom, Stasja Draisma, Johannes H. Smit

**Affiliations:** 1 Department of Research and Innovation, GGZ inGeest, Specialized Mental Health Care, 1081 HJ Amsterdam, The Netherlands; m.eikelenboom@ggzingeest.nl (M.E.); s.draisma@ggzingeest.nl (S.D.); jh.smit@ggzingeest.nl (J.H.S.); 2 Department of Psychiatry, Amsterdam Public Health Research Institute, Amsterdam UMC, Vrije Universiteit, 1081 HV Amsterdam, The Netherlands

**Keywords:** rapport, data quality, missing responses, socially desirable, consistency

## Abstract

The benefits of rapport between interviewers and respondents, in terms of recruiting the latter and motiving them to participate in research, have been generally endorsed. However, there has been less clarity with regard to the association between rapport and data quality. In theory, rapport could be beneficial if it motivates people to give complete and honest responses. On the other hand, efforts to maintain rapport by exhibiting pleasing and socially desirable behaviour could well be detrimental to data quality. In a large longitudinal epidemiological sample, generalized estimating equations (GEE) analyses were used to examine the association between rapport and the following three quality indicators: missing responses, responses to sensitive questions, and consistency of responses. The results of these analyses indicate an association between a high level of rapport and fewer missing responses. In contrast, we found more socially desirable responses for the high-rapport group. Finally, the high-rapport group did not differ from the low-rapport group in terms of the consistency of their responses.

## 1. Introduction

Research questions can be quite complex, and may involve sensitive topics. In such cases, face-to-face interviews are the best and, possibly, only way of collecting the data needed to answer such questions. In face-to-face settings, research data are obtained by means of a verbal interaction between the interviewer and respondent. In the case of survey designs in particular, the standardized interview is the golden standard to minimise the impact of this interaction on data quality. Even when such measures are taken, however, research has shown that interactions during interviews often have the potential to impact data quality, either in a positive or negative way [1,2,3,4]. Rapport is a key aspect of any interaction between two relative strangers, such as interviewers and respondents. Accordingly, rapport may be one of the mechanisms that can explain the impact of respondent and interviewer interaction on the quality of the data collected [5,6].

Rapport can be defined as a relationship that is built on mutual interest, support, and understanding. It is often viewed as an essential element of research assessments. This was already described more than sixty years ago, by Kahn and Cannell [7], who stressed that it is important for interviewers to show interest, support, and understanding. This motivates respondents to make accurate statements and to complete the interview. Interviewers often succeed in building rapport, even in highly structured and standardised interviews that present few opportunities to skip interview guidelines [8]; for example, interviewers can respond empathically, give compliments or advice, provide information, or even use humour, when appropriate. These types of behaviours foster the building of rapport. Other types of interviewer behaviour, such as sharing negative views on the study at hand or sharing too much personal information, are viewed as detrimental to rapport building.

Tickle-Degnen and Rosenthal [9] identify three components of rapport—mutual attentiveness, positivity, and coordination. In any interaction, mutual attentiveness and positivity are important ways of creating a positive personal image, which motivates the other person to continue the interaction. Coordination is described as the feeling that the interaction is balanced or “in sync”. In interview settings, all these three components are important. Mutual attentiveness and positivity help to keep respondents motivated to continue with the interview. Coordination facilitates smooth question and answer sequences throughout the interview. Garbanski, Schaeffer and Dykema [10] have created a definition of rapport, in line with the view that coordination is important in interview settings. They define rapport as responsive behaviour by the interviewer (“*fitting a response to the respondent’s previous task*”) and the respondent’s engagement *(“behaviours consistent with motivation to perform the task”).*

A common factor in these definitions is that rapport is viewed as a positive component of interactions in the research context, one that motivates people to participate, or to continue; for example, we have previously shown that good rapport at baseline has a beneficial effect on response rates in a subsequent wave of a longitudinal study [11]. Although it is generally acknowledged that rapport is important in interview settings, it is possible that rapport could have both a negative or positive effect on the quality of the data obtained. Good rapport may indeed be beneficial to data quality if it encourages respondents to give accurate and comprehensive answers. However, we could also argue that rapport can be detrimental to data quality if it promotes pleasing behaviour, both on the part of the interviewer and the respondent. In order to maintain rapport, the interviewer and respondent may consider it more important to present themselves in a favourable light during the interview than to give accurate answers to questions of a sensitive nature, or probe after such an answer, for example. This adverse effect of rapport is supported by Tourangeau and Yan’s [12] review of error in sensitive questions, which showed that respondents generally tend to give socially desirable answers to avoid causing embarrassment and offending the interviewer. 

Research so far has shown inconsistent results on the effect of rapport on data quality. In their research synthesis, West and Blom [5] analyzed eight studies on rapport and data quality. Five of these studies found a negative relationship between rapport and response quality, and the remaining three studies found no relationship. These differences in the findings might be explained by the diverse ways in which rapport and data quality are operationalized. The studies by Hill and Hall [13], Weiss [14], and Goudy and Porter [15] used interviewers’ post-interview evaluation ratings on topics such as being ill at ease or attitude towards the interview to measure rapport; whereas, a study by Van der Drift and Derksen [16] used interview style (formal, empathic, or intimate). Familiarity with the interviewer, based on previous waves of the study, was used as operationalization of rapport by Mensch and Kandel [17], while Belli, Lepkowski and Kabeto [18], Belli et al. [19], and Conrad et al. [20] created a rapport factor based on factor analyses of data concerning digression, laughter, and unacceptable feedback during the interview. Data quality was also operationalized in various ways, ranging from missing responses, to the number of interviews conducted by the interviewer (measuring interviewer performance), to responses that could be validated with existing data. 

Cordova Cazar [21], however, did find a positive association between rapport and data quality. Rapport was operationalized by using para data variables, such as a higher number of reported responses, a higher number of edits made to the responses, and choosing an open-response format over a programmed response more often. This study showed that high levels of rapport are associated with more complete responses and longer interview durations. These high levels boost respondents’ motivation to provide answers and to take sufficient time to do so. On the other hand, respondents tended to provide less additional detailed information when the rapport was good. The researchers ascribed this negative effect to the interviewers’ reticence to probe for more detailed information, prompted by their desire to avoid disturbing rapport and to keep the respondents motivated.

Olsen and Bilgen [22] showed that rapport between interviewers and respondents can potentially have an adverse effect on data quality. They found that more experienced interviewers obtain higher levels of acquiescence from respondents (the tendency to agree with questions without considering the question’s content). The authors argue that, as they gain experience, interviewers learn behaviours that may affect answering behaviour. More specifically, those verbal (or non-verbal) behaviours tend to increase rapport, causing the respondents to agree with experienced interviewers more often, to avoid offending the interviewer and disrupting the interaction.

Recently, Sun, Conrad and Kreuter [23] conducted an experiment in an effort to further clarify the relationship between rapport and data quality. They studied the effect of rapport, measured with an evaluation questionnaire on sensitive questions and item non-response. The results show a relationship between increased experience of rapport and disclosure on highly sensitive questions. A relation between rapport and the level of item non-response was not found.

While the studies described here show, in general, a relation between rapport and data quality, the results are ambiguous. The differences in the results may be explained by differences in the nature of the research setting, method, or domain in question; for example, some studies operationalise rapport by coding and interpreting the verbal behaviour of interviewers and respondents during interviews. In other studies, interviewers or respondents are asked to judge rapport by filling out an evaluation questionnaire on the perceived rapport after the interview. Furthermore, the evaluation of rapport is most often one sided, either from the perspective of the interviewer or the perspective of the respondent. However, rapport results from the interaction between the interviewer and respondent, as the definition of Garbanski, Schaeffer and Dykema [10], for example, illustrates. To incorporate both the contribution of the respondent as well as the contribution of the interviewer to the establishment of rapport, we will explore the relation between data quality and rapport using evaluations on rapport from both. 

The aim of this study is to elaborate on previous studies and contribute to the knowledge on this challenging topic, using data from the Netherlands Study of Depression and Anxiety (NESDA), an epidemiological longitudinal cohort study with a survey design. Interviewer and respondent evaluations have been part of the interview procedures since the start of the study. Working with these data provides the opportunity to study the effect of rapport in a setting that is a reflection of realistic research procedures, in a naturalistic observational design. Hopefully, this leads to more insights into the role of rapport on data quality that can be used in studies with similar research procedures. We analyse the effect of rapport on the quantity of missing responses, on socially desirable responses to sensitive questions, and on the consistency of responses. 

## 2. Materials and Methods

### 2.1. Sample

The sample used for the purpose of this study consisted of the Netherlands Study of Depression and Anxiety (NESDA) cohort, which started in 2004. NESDA is an ongoing longitudinal study on the development and course of depression and anxiety disorders. Individuals with a current depression and/or anxiety disorder (*n* = 1701), a past diagnosis or with subthreshold symptoms (*n* = 907), and healthy controls (*n* = 373) were recruited for the study. Respondents aged 18–65 were included. The recruitment of respondents in the Netherlands took place in Amsterdam, Groningen, and Leiden, from September 2004 to February 2007. Respondents were recruited from the general population (*n* = 564), primary care (*n* = 1610), and specialised mental health care (*n* = 807). For more details concerning the design used and the procedures followed, see Penninx et al.’s study [24]. The study protocol was approved by the ethical review boards of all the participating centres, and all the participants gave their written informed consent. Any respondents with a primary clinical diagnosis of a psychotic disorder, obsessive compulsive disorder, bipolar disorder, or an addiction disorder were excluded. The same applied to any respondents who were not able to communicate in Dutch. The NESDA sample aimed to be representative of a psychiatric population in routine mental health care in the Netherlands, with healthy controls added. For this study, respondents who had not completed the evaluation form were excluded from the analyses because rapport could not be measured in these cases. The remaining sample consisted of 971 male participants (33.5%, mean age of 43.4) and 1926 female participants (66.5%, mean age of 41.0). Ninety-seven percent of the sample had a Dutch nationality. To measure consistency, we used a question on alcohol use. All the respondents in the sample who reported alcohol use were selected for analysis, creating a subsample of 2371 respondents, consisting of 844 males (35.6%, mean age of 43.5) and 1527 females (64.4%, mean age of 40.6). It was found that 98.1% of these respondents had a Dutch nationality. 

### 2.2. Baseline Assessment

The respondents were invited to attend a morning session at one of the Dutch clinical sites (Amsterdam, Groningen, and Leiden), for a face-to-face assessment. During these assessments, computer-assisted structured interviews were used to gather information on psychopathology and demographic characteristics, as well as on physical and psychosocial functioning. This assessment also included medical measurements, computer tasks, and an evaluation of the interview by both the respondents and the interviewers. On average, the assessment took four hours to complete. In addition to this face-to-face assessment, the respondents filled out two self-administered questionnaires at home. Data from the baseline assessment were chosen for analyses because this was the first opportunity to build rapport between the respondent and interviewer. Rapport during follow-up waves of the study might be affected by rapport that was built during previous waves, making the results of the effect of rapport on data quality less clear to interpret.

### 2.3. Recruitment and Training of Interviewers

The interviewers, who were recruited via advertisements, were required to have an intermediate vocational educational qualification/community college-level qualification at the very least. They also needed to have good social skills, affinity with the study population, and, preferably, experience in conducting semi-structured interviews. In total, 47 interviewers were recruited at the start and during the baseline assessment.

The newly recruited interviewers were given extensive training by the fieldwork coordinator (a five-day course) on how to conduct the NESDA assessment, together with a detailed training manual. This training course mainly focused on how to conduct the assessment in a standardised manner. During the fieldwork period, interviews were recorded and these recordings were used to give feedback to the interviewers on their performance. This procedure was put in place to ensure that all interviewers adhered to the interview protocol for standardised interviewing, and thus collected high-quality data. In addition, regular meetings were held with the interviewers to discuss key assessment-related topics, such as difficulties with the assessment itself and any related questions. The goal was to standardise interview behaviour across the interviewers.

### 2.4. Measurements

#### 2.4.1. Rapport 

To capture the dyadic character of rapport, at the end of the assessments, respondents as well as interviewers filled in an evaluation form concerning their interview experience. Both the interviewers and the respondents were asked to judge the extent to which they had enjoyed the interview experience. We argue that a mutually pleasant experience reflects a high level of rapport between the interviewer and the respondent, given that the NESDA study itself focuses on depression and anxiety, which are generally not considered to be pleasant topics to discuss.

The question for the respondents was formulated as follows: *“How do you rate the interview?”* The responses were measured on a 4-point scale, ranging from ‘pleasant’ to ‘unpleasant’. The interviewers were asked, *“Overall, how did the interview with the respondent go?”* The response scale for the interviewers was more extensive, also containing a very pleasant and very unpleasant category, making it a 6-point scale. To make the responses comparable, we created a dichotomous variable. To achieve this dichotomous variable, response options were first divided into a high-rapport category (very pleasant, pleasant and a bit pleasant) and low-rapport category (neither pleasant nor unpleasant, unpleasant and very unpleasant). Next we compared the interviewer responses with the respondent responses. We judged rapport to be high when both the interviewer and the respondent rated the interview as pleasant. When the interviewer and the respondent ratings differed, or both were rated as low, we judged rapport to be low. 

#### 2.4.2. Data Quality

To study the relationship between rapport and data quality, the association between rapport (as described in the previous paragraph) and three quality indicators was measured. These quality indicators were chosen based on the availability in the NESDA study. They are as follows:

1. *Missing responses.* A computer-assisted personal interview (CAPI) was used for the purposes of the NESDA study. The CAPI does not allow questions to be skipped, so the interviewer has to make a conscious decision to record an answer as missing. Due to the use of CAPI, the total amount of missing responses per interview was expected to be low. The following two categories were created: ‘one missing responses or none’ and ‘two or more missing responses’. To this end, a sum score was created for missing responses using 678 questions in total on the following topics: demography, medication, health, healthcare use, childhood trauma, important negative events, and suicidal behaviour. These particular topics from the NESDA interview were selected because the associated questions were mandatory for all the respondents, whereas those involving other topics (such as depression, anxiety, manic disorder, and alcohol use) were only answered by a subgroup of respondents (missing by design).
2. *Socially desirable responses.* We measured differences in the distribution of responses to sensitive questions, since socially desirable responses are expected for questions of a sensitive nature. To this end, we selected questions on topics that had been reported to be sensitive. For a topic to be considered sensitive, one of the following criteria must apply: the question must be intrusive, the respondents must have concerns about the consequences of answering such questions honestly, or the question must elicit answers that are perceived to be socially undesirable [12]. The following questions meet these criteria:
  *Income 1: “In general, do you have enough money to buy the food that you and your family need?” (yes/not always)*
  *Income 2: “At the end of the month, do you have money left, do you have just enough money or do you not have enough money?”(yes, enough/no, not enough)*
  *Income 3: “Have you ever experienced serious financial difficulties?” (no/yes)*
  *Misdemeanour 1: “Have you ever had dealings with the police or the courts in connection with a misdemeanour?”(no/yes)*
  “Yes” responses to the first and second questions about income were defined as socially desirable, as were “no” responses to the third and fourth questions. Socially desirable responses were all coded as “0” and used as the reference category in the analyses.
3. *Consistency of response.* Answers to a comparable question—in two data collection modes—were compared, to measure consistency of responses. The respondents were asked to fill in a questionnaire at home before attending a face-to-face interview. The following questions were used in the self-report questionnaire (first question) and in the face-to-face interview (second question):
  *“When you drink, how many glasses of alcohol do you drink on a typical day?” (self-assessment)1 – 2, 3 – 4, 5 – 6, 7 – 9, or 10 or more*
  *“On the days that you used alcohol in the past 12 months, how many glasses would you typically drink in one day?” (face-to-face assessment)…….. alcoholic beverages*
  Responses to the face-to-face interview were classified according to the ordinal categories used in the self-report questionnaire. Next, the responses to both questions were compared by creating the following two categories: 1. Same response to both questions; 2. Different response to both questions.

### 2.5. Statistical Analyses

Analyses were performed using IBM SPSS Statistics 24 (IBM Corporation, Armonk, NY, USA). First, we performed chi-square tests to analyse the association between the level of rapport and the following demographic variables: gender, age, and level of education. Some interviewers are probably better able to build rapport than others. Therefore, interviews conducted by the same interviewer cannot be viewed as independent measures, but are instead nested within that interviewer. To handle our nested data, we chose to perform a generalized estimating equations (GEE) analysis. GEE analysis is able to handle the non-normally distributed nested data of this study and is a relatively simple and direct method to use [25]. GEE analysis provides parameter estimates and accurate standard errors even when the correlation structure is not perfectly specified. Since the outcome variable was dichotomous, a logistic binomial GEE analysis was conducted to analyse the relationships between rapport and the data quality indicators.

## 3. Results

The demographic characteristics of the respondents, such as age, gender, and education, are likely to be associated with data quality. We adjusted for these characteristics in our main analyses. Unfortunately, we were not able to adjust for western vs. non-western descent, since 97% of our sample was of western descent. Western descent was based on the nationality and country of birth of the participant (if these were both from the Netherlands, other European countries (excluding Turkey), the USA, or Canada). Only 112 participants were of non-western descent, coming from 17 different countries, making these groups too small to consider for analyses. Table 1 lists the demographic variables for the respondents who participated in the NESDA study. We found no gender-based differences in the level of rapport, nor any differences between respondents with different levels of education. However, our results do indicate an association between age and rapport. 

Table 2 shows the distribution of the variables used in the GEE analyses. As expected, we found low frequencies of missing responses due to the study process design, in which missing responses are actively prohibited. 

Table 3 shows the results of the GEE analyses of associations between rapport and our quality indicators.

### 3.1. Missing Responses

We found an effect of rapport on the number of missing responses. The odds ratio of more missing responses is 0.8 in the group with high levels of rapport. This indicates that a high level of rapport decreases the odds of more missing responses by 20%. This result remained significant after adjusting for gender, age, and level of education.

### 3.2. Socially Undesirable Responses

The results also indicated a significant relation between rapport and socially desirable responses to sensitive questions. The odds of respondents reporting not having enough money to buy food was 0.65 in the high-rapport group. The odds of reporting not having enough money left over at the end of the month was 0.62 when there was a high level of rapport. Reporting severe financial problems also occurred less often in the high-rapport group, with an odds ratio of 0.70. Finally, the group with a high level of rapport reported misdemeanour-related dealings with the police or the courts less often, with an odds ratio of 0.72. These results indicate that the respondents in the high-rapport group tended to give more socially desirable responses.

### 3.3. Consistency of Response

Finally, we examined the consistency of the responses by comparing a question from the self-report questionnaire to a question from the face-to-face interview. Here, we found no significant difference between high levels of rapport and low levels of rapport in consistently reporting the amount of alcohol intake.

## 4. Discussion

The results showed that rapport between the interviewers and the respondents was associated with measures of data quality. However, the direction of the effects that we found differed depending on which measure of data quality was analysed. Missing responses were less likely to occur in cases where there were high levels of rapport. This finding is in line with previous studies, which indicated that rapport seems to promote more complete answers [5,21]. Rapport seems to motivate respondents to cooperate with the interviewer, thereby preventing missing data.

Besides this positive effect of rapport on data quality, the results also showed a negative effect of rapport on data quality. When there is a high level of rapport, respondents are less likely to respond to sensitive questions in a socially undesirable and, therefore, honest manner. As previously stated, Tourangeau and Yan’s [12] paper indicates that participants generally give socially desirable answers to avoid embarrassment, and to avoid offending the interviewer. In that light, our finding does not seem surprising. When building rapport, or when it has already been established, respondents want to protect the existing level of rapport by avoiding any responses that might cause embarrassment or offense.

Finally, we studied the effect of rapport on the consistency of answers by comparing a question from the self-report questionnaire with a question posed during the face-to-face interview. We found that rapport has no significant effect on the consistency of the responses. One factor that might account for this result is the type of question used in the analysis. In general, people do not tend to track their exact alcohol intake during their day-to-day lives. Instead, they may need to make estimates based on the information recalled from their memory. Where this is the case, memory might play a more important role in recalling consistent information than rapport.

The study has some limitations. Firstly, the interview deliberately involved the use of a computer-assisted personal interview, which was programmed to prevent missing responses. Also, the interviewers were instructed to never skip questions. The only way that interviewers could circumvent this, and actually skip questions, would be by consciously deviating from the protocol. As a result, there was a very low number of missing responses. Therefore, in a situation where the interviewers are not actively discouraged from skipping questions, the results might well be different.

Secondly, it is possible that the evaluations used to create the rapport variable are susceptible to socially desirable responses. The respondents were asked to fill in an evaluation form and hand it back to the interviewer. This procedure could cause the respondents to modify their response, to some extent, to avoid offending the interviewer. If so, then the number of instances of high-level rapport might be an overestimation.

Finally, we should mention that our results might be limited in their generalizability, due to the characteristics of our sample. The respondent sample used in this study is representative of a psychiatric population of predominantly western descent, in routine mental health care. Also, the sample of interviewers for this study should be taken into consideration. Although we selected 47 interviewers from three regions in the Netherlands, the majority of the interviewers who were active in this research were female. Furthermore, because of the design, the workload of the interviewers was not balanced, and it is possible that certain interviewers contributed more to the results than others. The interpretation of the findings should be handled with care. 

Despite these limitations, the large sample size of the study and number of face-to-face interviews, and the rich data collection of the NESDA study made it possible to study the effect of rapport on different measures of data quality. Also, the large number of evaluations filled in by both the interviewer and the respondent enabled us to create a measure of rapport based on the actual shared experience. Although most studies use evaluation from either the respondent or the interviewer, this study took both views into account, providing insights into the complex topic of the relationship between rapport and data quality. 

## 5. Conclusions

It is clear that rapport plays a part in studies that collect data through face-to-face interactions between interviewers and respondents. In general, interviewers vary in their communication skills and their ability to build rapport. Therefore, it is important to hire interviewers with good communication skills, and further train them in rapport building skills.

However, our results confirm that it is difficult to clearly identify the effect of rapport on specific aspects of data quality. As West and Blom [5] suggest in their research synthesis, this can be explained by the lack of clear definitions and operationalisations of rapport, which tends to hamper any study of rapport and data quality. Future research might benefit from a standardised rapport measurement questionnaire for interviewers and respondents, to make studies more readily comparable, and to produce clearer results.

## Figures and Tables

**Table 1:** Demographics of the NESDA respondents.

General Sample		Subsample for Consistency Variable	
Variable	High Level of Rapport (%)	Variable	High Level of Rapport (%)
Gender		Gender	
*Female (n = 1926)*	84.6	*Female (n = 1527)*	84.9
*Male (n = 971)*	82.5	*Male (n = 844)*	82.5
Age ***		Age ***	
*18–24 (n = 391)*	93.2	*18–24 (n = 327)*	93.3
*25–39 (n = 831)*	86.4	*25–39 (n = 668)*	87.0
*40–49 (n = 689)*	83.1	*40–49 (n = 568)*	83.1
*50–65 (n = 986)*	78.4	*50–65 (n = 808)*	78.6
Level of education		Level of education	
*Basic (n = 192)*	78.0	*Basic (n = 126)*	77.8
*Intermediate (n = 1695)*	84.5	*Intermediate* *(n = 1350)*	84.8
*High (n = 1010)*	83.8	*High (n = 895)*	83.8

Using a chi-square test, *** *p* < 0.001.

**Table 2:** Distribution of dependent and outcome variables.

Variable	%
**Rapport** *Low (n = 483)* *High (n = 2414)* *Missing (n = 0)*	16.7 83.3 0
**Total no. of missing responses** *One or none (n = 2090)* *Two or more (n = 807)*	72.2 27.8
**Socially desirable responses** Income 1 Enough money to buy food? *Yes (n = 2611)* *No (n = 286)* *Missing (n = 0)*	90.1 9.9 0
Income 2 Enough money at the end of the month *Yes (n = 2559)* *No (n = 336)* *Missing (n = 2)*	88.3 11.6 0.1
Income 3 Ever had serious financial problems? *No (n = 2190)* *Yes (705)* *Missing (n = 2)*	75.6 24.3 0.1
*Ever had dealings with police or court?* *No (n = 2405)* *Yes (n = 491)* *Missing (n = 1)*	83.0 17.0 0.0
**Consistency of response** Discrepancy in report of alcohol intake face to face interview vs. self-report * *Inconsistent response (n = 1555)* *Consistent response (n = 816)* *Missing (not applicable)*	34.4 65.6

* A subsample of all 2371 respondents who used alcohol was used for this variable.

**Table 3:** Association between rapport and data quality assessed with GEE analysis.

	Beta Estimate	Std Error	95% CI	Odds Ratio
Total no. of missing responses	−0.225*	0.089	(−0.400, −0.050)	0.798
socially desirable responses Income 1 Income 2 Income 3 Problems with the law	−0.426 ** −0.479 ** −0.356 ** −0.323 *	0.147 0.140 0.105 0.155	(−0.715, −0.138) (−0.752, −0.0205) (−0.562, −0.150) (−0.626, −0.019)	0.653 0.620 0.701 0.724
Consistency in reporting alcohol intake – F-t-F vs. self-report	−0.133	0.100	(−0.329, 0.062)	0.875

Table 3. Association between rapport and data quality assessed with GEE analysis, * *p* < 0.05, ** *p* < 0.01.

## Data Availability

In our analyses, we used data from the NESDA study. NESDA fully adheres to best-practice FAIR data principles (i.e., NESDA data are findable, accessible, interoperable, and reusable). Available data can be located via a public data listing resource, which can be found on the following website: www.nesda.nl (accessed on 12 October 2021). Based on this, researchers can file a data request. When access is granted, after review, data can be downloaded from the central NESDA data repository. These data files are available in a standard data format (SPSS), ready for further analysis. Finally, the reusability of the NESDA data, and the commitment of the NESDA consortium to long-term data stewardship, is illustrated by the large number of scientific articles that have been published in the past two decades.
